# Visualization and characterization of RNA–protein interactions in living cells

**DOI:** 10.1093/nar/gkab614

**Published:** 2021-07-27

**Authors:** Ningjun Duan, Maria Arroyo, Wen Deng, M Cristina Cardoso, Heinrich Leonhardt

**Affiliations:** Department of Biology II, Ludwig Maximilians University Munich, Munich 81377, Germany; Department of Oncology, The First Affiliated Hospital of Nanjing Medical University, Nanjing 210029, China; Department of Biology, Technical University of Darmstadt, Darmstadt 64287, Germany; Department of Biology II, Ludwig Maximilians University Munich, Munich 81377, Germany; College of Veterinary Medicine, Northwest A&F University, Yangling 712100, China; Department of Biology, Technical University of Darmstadt, Darmstadt 64287, Germany; Department of Biology II, Ludwig Maximilians University Munich, Munich 81377, Germany

## Abstract

RNA–protein interactions are the structural and functional basis of significant numbers of RNA molecules. RNA–protein interaction assays though, still mainly depend on biochemical tests *in vitro*. Here, we establish a convenient and reliable RNA fluorescent three-hybrid (rF3H) method to detect/interrogate the interactions between RNAs and proteins in cells. A GFP tagged highly specific RNA trap is constructed to anchor the RNA of interest to an artificial or natural subcellular structure, and RNA–protein interactions can be detected and visualized by the enrichment of RNA binding proteins (RBPs) at these structures. Different RNA trapping systems are developed and detection of RNA–protein complexes at multiple subcellular structures are assayed. With this new toolset, interactions between proteins and mRNA or noncoding RNAs are characterized, including the interaction between a long noncoding RNA and an epigenetic modulator. Our approach provides a flexible and reliable method for the characterization of RNA–protein interactions in living cells.

## Graphical Abstract

**Graphical Abstract:**
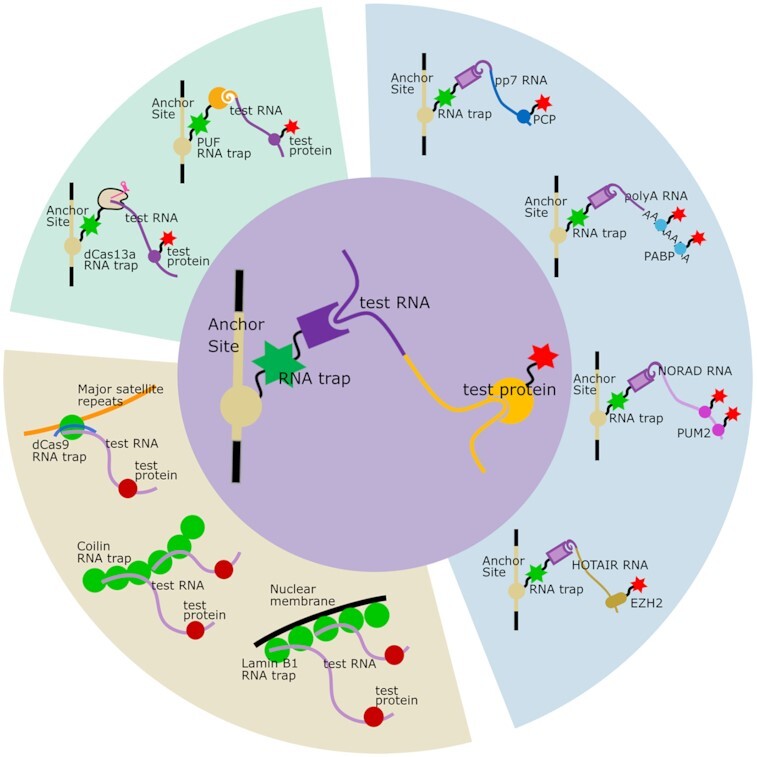
The basic principle, applications and extensions of RNA trap based RNA-protein interaction assay.

## INTRODUCTION

The RNA in cells is commonly associated with RNA binding proteins (RBPs), which is required for the proper function of both RNAs and proteins. On the one hand, the processing, transport, function and stability of RNAs are modulated by the RBPs, e.g. mRNA processing and ribosome assembly require different groups of RBPs to accomplish these biological processes (1–3). On the other hand, proteins can also be functionally modulated by the binding of RNAs. A typical example is the widely applied CRISPR/Cas9 genome editing technique, in which binding of a guide RNA (gRNA) to the Cas9 protein modulates the conformation of Cas9 protein and activates its endonuclease activity on the targeted DNA sequences (4–7). Therefore, identification and characterization of the physical interactions between RNA and protein is the basis for revealing the function of RNAs and RBPs.

Being physically flexible and biochemically unstable, this intrinsic property of RNA molecules makes it difficult to identify the interaction between RNAs and proteins. Traditional approaches like electrophoretic mobility shift assay (EMSA) require purification of RNAs as well as proteins to identify their physical interaction by electrophoresis *in vitro*, which is *in praxis* complicated and with limited throughput. Other biochemical methods like immunoprecipitation, cross-linking and proximity-labeling, in combination with high throughput sequencing or mass spectrometry (8–11), have been developed and massively applied for screening of RNAs binding to protein or proteins binding to RNA, but it is still challenging to visualize and characterize the interaction inside living cells. Recently developed RNA visualization techniques, using fluorescent RNA aptamers (like Spinach, Broccoli etc.) (12,13) or RNA tags bound by specific proteins (like ms2, pp7 and λN22 etc.) (14–17), allow for imaging RNAs or RNA translation in cells. One of the most frequently used RNA tags, the bacteriophage ms2 RNA hairpin structure, which is specifically bound by the MS2 coat protein (MCP), is fused to RNAs of interest (ROIs), and the tagged ROIs can thus be visualized by the fluorescently labeled MCP. It is, however, still challenging to image RNA–protein interaction directly in cells as the detection is limited by the abundance of RNAs or proteins, as well as the generally low binding affinities between them (18).

To overcome these limitations, here we introduced a RNA fluorescence three-hybrid (rF3H) method for RNA–protein interaction analyses in cells. In this method, RNA molecules are recruited and anchored at specific subcellular structures by a designed RNA trap, and the interaction between the trapped RNAs and fluorescently labeled RBPs is visualized and identified via fluorescence co-localization at these subcellular structures. With this new method, we measured the interactions between proteins and different types of RNAs, and in particular studied the interaction between an epigenetic factor EZH2 protein and the HOTAIR non-coding RNA (ncRNA). Different RNA trapping systems and multiple cellular anchoring structures were also explored for broad applications of this tool. Our study established a fluorescence hybrid assay in mammalian cells, providing a flexible and reliable approach for the characterization of RNA–protein interactions.

## MATERIALS AND METHODS

### Plasmids

The information of plasmids constructed in this study was shown in Supplementary Figure S1, and the source of the fragments used were listed in Supplementary Table S1. In Generally, the MCP RNA trap plasmid pMCP-EGFP-LacI was constructed by replacing the GFP binder of pGBP-LacI plasmid (19) with ms2 coat protein (MCP) and enhanced green fluorescent protein (EGFP) open reading frames (ORF). All the other RNA trap plasmids were constructed on pMCP-EGFP-LacI. To construct RNA traps anchoring to the nuclear envelope, Cajal bodies, and genome loci, the LacI coding part of pMCP-EGFP-LacI was replaced by the ORFs for Lamin B1, Coilin and dCas9, respectively. The MCP was replaced by the artificially designed PUF domain and Lbu-dCas13a to get PUF and dCas13a mediated RNA traps.

The CMV cassette test RNA plasmids were developed on pEGFP-N1 (Clontech). The whole EGFP ORF was firstly replaced with 6 times of ms2 stem–loop sequences to construct the ms2 RNA plasmid. After that, 4 of 6 times ms2 stem–loops were replaced by 4 times of wildtype or mutant (GGAGCAGACGATGGCGTCGCTCC, synthesized by Eurofins) pp7 stem–loops, whole-length NORAD, HOTAIR 1–300 and its shortened fragments, as well as NORAD/HOTAIR 201–300 hybrid fragment, to get the respective ms2 tagged RNA plasmids. The PUF recognition sequence-tagged RNAs were constructed by inserting a 9-nt sequence (TGTTGTATA) to the 3′ end of ms2, pp7 and HOTAIR 1–300 sequences of their plasmids. And the U6 cassette ms2-pp7 RNA plasmid was constructed by cloning the RNA sequence from its CMV cassette plasmid into the U6 cassette of the pEX-A-u6 plasmid (20).

The test protein plasmids were also derived from pEGFP-N1. The EGFP fragment was replaced by mCherry ORF in the beginning, followed by cloning the ORFs coding for MCP, PCP, PABPC1, PUM2, EZH2, EZH2N (1–370 amino acids of whole-length EZH2 protein), EZH2C (371–751 amino acids of whole-length EZH2 protein) and the mutant proteins (MCP S47R, EZH2N T350A and EZH2N T350D) with a N-terminal nuclear localization sequence (NLS) to the upstream of mCherry. The mScarlet tagged PCP protein was constructed by replacing the mCherry ORF of the PCP-mCherry protein with a mScarlet-I ORF.

### Cell culture, transfection and manipulation

BHK cells containing a genomic integration of multiple *lac*O sites (21) and HeLa cells were cultured in modified Eagle's medium (DMEM, Sigma) supplemented with 10% fetal bovine serum (FBS, Sigma) and 10 μg/ml gentamicin (Thermo Scientific); mouse J1 embryo stem cells were cultured in DMEM supplemented with 16% fetal bovine serum, 10 U/ml penicillin/streptomycin, 2 mM l-glutamine, 0.1 mM β-mercaptoethanol, 1 μM PD0325901, 3 μM CHIR99021 and 1000 U/ml LIF, 1× non-essential amino acid (NEAA, Thermo Scientific). All the cells were incubated at 37 °C in a humidified environment with 5% CO_2_.

Transient transfection was performed with Lipofectamine 3000 (Thermo Scientific) following the manufacturer's instruction. For one well of a six-well plate, in total 2.4 μg plasmid DNA (with the mass ratio of RNA trap: ROI: POI = 0.8 μg: 0.8 μg: 0.8 μg) was used for transfection. For transfection, 4 μl of Lipofectamine 3000 was diluted in 120 μl Opti-MEM (Thermo Scientific) in one tube and incubated for 5 min at room temperature. The three plasmids for rF3H (2.4 μg) as well as 4 μl of P3000 were diluted and mixed in another tube with 120 μl Opti-MEM. The contents of both tubes were then mixed gently and incubated for 10 min at room temperature. The mixtures were then added to cells drop by drop and the cells were put back into the incubator overnight.

For fixed cell imaging, cells were seeded on 18 mm × 18 mm coverslips. About 24 h after transfection, cells were firstly fixed with 3.7% formaldehyde in PBS for 10 min. The fixed cells were then stained with 1 μg/ml DAPI in PBS directly and then mounted onto slides with Vectashield mounting medium (Vector Laboratories).

For immunofluorescence, cells were fixed as described before and permeabilized with 0.25% Triton X-100 in PBS for 5 min, blocked with 1% BSA for 30 min, incubated with BSA diluted mouse anti-HA primary antibody (Abcam, ab18181) and Alexa Fluor 594 labeled donkey anti-mouse IgG secondary antibody (Abcam, ab150108), then counterstained with DAPI, and the samples were mounted as described above.

For RNA fluorescence *in situ* hybridization (FISH), a set of Cy5 labeled single stranded oligonucleotide probes (CTCTGCTGGTTTGTACAATC, AATGAACCCGGGAATACTGC, AGGAATTAGG TCCTTAGG, ATATCGTCTGCTCCTTTCTG, synthesized by Eurofins) that target pp7 sequence was used. After fixation and permeabilization of the cells, FISH was processed according to Vidisha's protocol (22).

For live-cell imaging, cells were pre-plated on an 8-wells μ-Slide (ibidi) 1 day before transfection. Nuclear staining was performed by adding 1μM SiR-DNA (Cytoskeleton) 30 min before imaging.

### Fixed and live-cell imaging and quantification

Both fixed and live-cell imaging were carried out with an SP8 confocal microscope (Leica). A 405 nm diode laser was used for DAPI excitation, while the 488, 561, 594 and 647 nm beams from a 470–670 nm white laser were used for the excitations of EGFP, mCherry, Alexa Fluor 594, Cy5 and SiR-DNA. The emission of GFP was detected by a PMT sensor while the other three emissions were all received by HyD sensors. A 63x oil objective was chosen for imaging, and a sequential imaging method, which detects the fluorescence of DAPI, EGFP, mCherry, Alexa Fluor 594, Cy5 or SiR-DNA individually, was set as the default scanning method. Image analyses were performed with LAS X and ImageJ software.

As shown in Supplementary Figure S2, to quantify the relative fluorescence at *lac*O spots, the signal from DAPI channel was applied to partition the area of nucleus at first, the mean fluorescence intensities (average gray values) of the *lac*O spot and the whole nucleus from EGFP channel were calculated as *Green_lacO_* and *Green_nucleus_*, respectively, and then the same process was utilized to get *Red_lacO_* and *Red_nucleus_* from mCherry channel. Both green and red signals of the whole nucleus were applied to normalize variation in expression levels in cells. The relative fluorescence at the *lac*O spots was calculated as follows.

$$\begin{equation*}\frac{{Re{d_{lacO}} - Re{d_{nucleus}}}}{{Gree{n_{lacO}} - Gree{n_{nucleus}}\ }}\end{equation*}$$

For each experiment, the relative fluorescence was normalized by the control group without RNA.

### RNA extraction and quantitative PCR

Nuclear extraction was performed as follows. Cells were detached by trypsin and harvested by centrifugation Cells were washed twice with cold PBS and the pellet was gently resuspended in 500 μl 1x hypotonic buffer (20 mM Tris–HCl, pH 7.4, 10 mM NaCl, 3 mM MgCl_2_) by pipetting up and down several times and incubated on ice for 15 min. 20 μl detergent (10% NP40) was added and vortexed for 10 s. The homogenate was centrifuged for 10 min at 3000 rpm and at 4°C. The pellet corresponded to the nuclear fraction. The total and nuclear RNA were extracted with the NucleoSpin RNA kit (MACHEREY-NAGEL) following the manufacturer's instructions, and the synthesis of cDNA was performed with High-Capacity cDNA Reverse Transcription Kit (Applied Biosystems) as instructed by the manufacturer. The expression level of the ms2-pp7 RNA under the control of CMV and U6 cassettes were quantified by real-time PCR with the primers (Forward: 5′ ATATCTGCAGGTCGACTC 3′, Reverse: 5′ CTGCTCCTTTCTGAATTCC 3′).

### Statistics

Student's t-test was applied for the experimental data. *P* < 0.05 was chosen as the limit of significance, and marked as * *P* < 0.05, ** *P* < 0.01, *** *P* < 0.001. All the relative fluorescence data were presented as scatter plots with arithmetic means ± standard deviations.

## RESULTS AND DISCUSSION

### Development of the RNA fluorescence three-hybrid (rF3H) assay

**Figure 1.**
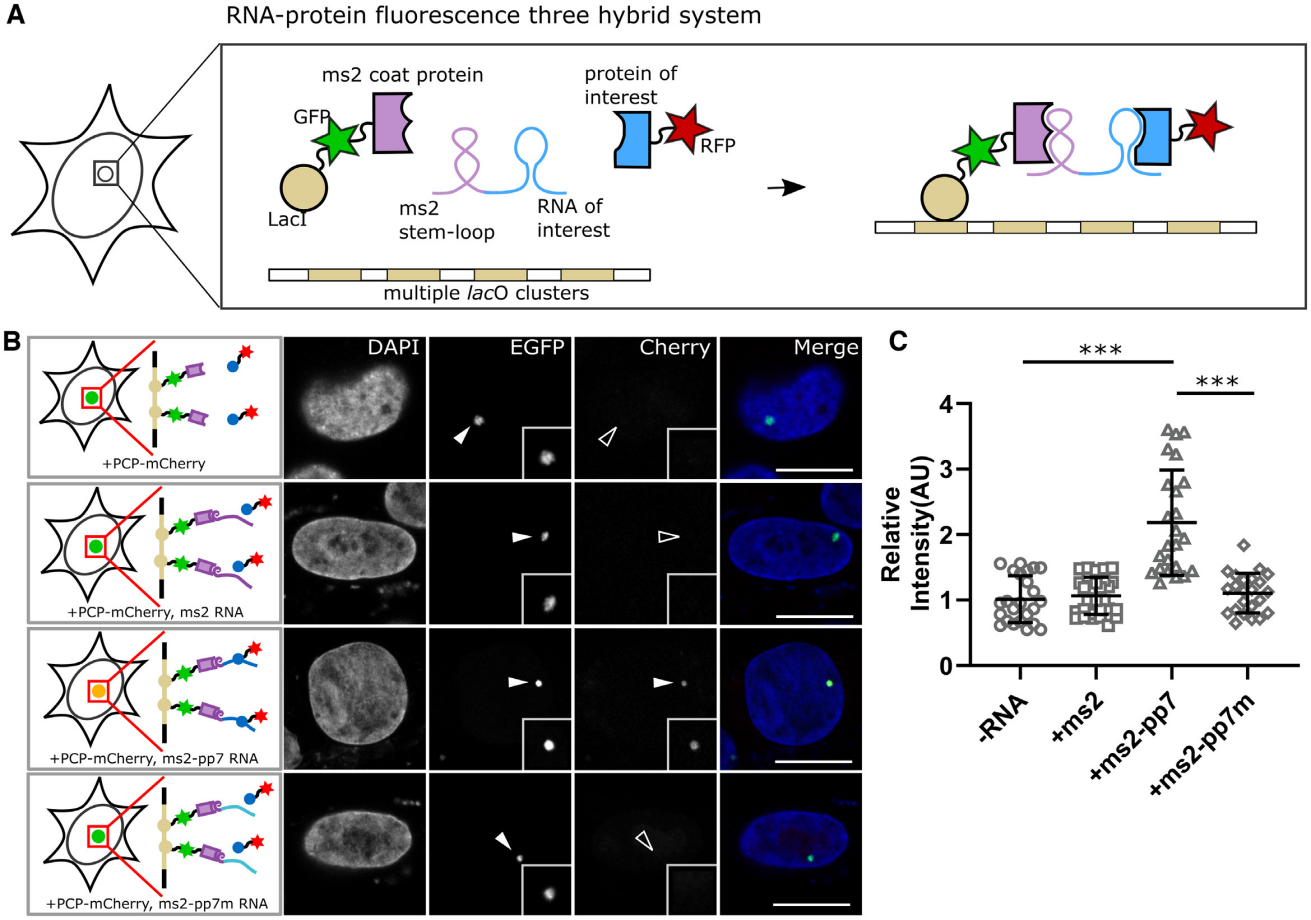
Development of the RNA fluorescence three-hybrid assay (rF3H) for RNA–protein interaction studies. (**A**) Principle of the rF3H assay. Three components for the assay, RNA of interest, protein of interest and an RNA trap, are triply expressed in cells containing lacO array on the genome. Tagged with ms2 stem–loop structures, the RNA of interest is recruited and anchored to the lacO loci by the RNA trap, the protein of interest therefore is enriched at the lacO loci by interacting with the RNA of interest, and the interaction between the protein and RNA can be visualized as co-localization of the fused red and green fluorescent proteins. (**B**) Image examples of pp7 RNA-PCP interaction visualized by rF3H. The RNA trap marked by EGFP was anchored at the lacO array and visualized as a nuclear fluorescent spot (EGFP channel, marked by filled arrowhead), and the mCherry labeled PCP (PCP-mCherry) was recruited to the lacO spot specifically in the presence of pp7 RNA (lower panel, marked by filled arrowhead), but neither in the mutant pp7 group nor in the control group (mCherry channel of the upper and middle panels, marked by open arrowhead). Merge channels were enhanced for clarity purposes. Scale bars are 10 μm. (**C**) Quantification of the relative mCherry signal at the lacO array. The presence of pp7 RNA leads to a significant fluorescence enhancement at the lacO spot compared with no RNA or only ms2 RNA condition. Data are presented as mean ± S.D., -RNA, *n* = 23; +ms2, *n* = 25; +ms2-pp7, *n* = 24, +ms2-pp7m, *n* = 26. *** *P* < 0.001.

To visualize RNA–protein interactions, we designed an RNA fluorescence three-hybrid (rF3H) assay (Figure 1A). In this assay, a RNA trap, which consists of a MS2 coat protein (MCP), a Lac repressor (LacI), and an EGFP, is used to capture RNAs onto a bacterial *lac* operon (*lac*O) array that is integrated into the genome of a mammalian cell line. The MCP binds the ms2 stem–loop tagged RNA of interest (ROI) and anchors the RNAs at the genomic *lac*O loci via the fused LacI protein. This RNA trap, together with the trapped RNA of interest, is visualized as a fluorescent spot in cell nuclei via EGFP under a fluorescence microscope. The potential RNA binding protein (referred as protein of interest, POI) tagged with a red fluorescent protein (e.g. RFP or mCherry) is recruited to the *lac*O array by interacting with the trapped ROIs, and the RNA–protein interaction thereby can be identified by co-localization of the green and red fluorescence at the nuclear *lac*O spot.

As a proof-of-principle, we tested this rF3H strategy with a well-characterized RNA–protein interaction pair. We tagged the pp7 RNA with ms2 stem–loops and attempted to visualize the interaction between pp7 RNA and the pp7 coating protein (PCP). We tested the interaction between pp7 and PCP protein. When triply expressed in cells containing *lac*O array, the RNA trap together with the trapped pp7 RNA was clearly imaged as a nuclear spot marked by EGFP fluorescence. The enrichment of the mCherry-PCP at the *lacO* loci was also exclusively detected in the presence of pp7 RNAs, but not with the RNA trap itself, irrelevant RNAs and the mutant pp7 RNA (Figure 1B), which indicates an interaction between pp7 RNA and PCP protein. Quantification of the relative fluorescence intensity showed a significant (about two times higher) enrichment of PCP-mCherry at the lacO spot resulted by pp7-PCP interaction (Figure 1C), demonstrating the feasibility of this rF3H strategy for RNA–protein interaction detection. We applied RNA FISH with anti-pp7 probes to confirm the capture of ROI, as well as IF with anti-HA antibody to verify the aggregation of POI (Supplementary Figure S3A, B), which further demonstrates the feasibility of this method.

### Optimization of the rF3H assay

To detect RNA–protein interactions sensitively and precisely, we optimized the stoichiometry for RNA trap and POI used in the rF3H assay. With a constant amount of plasmid for ROI transcription, we adjusted the amounts of plasmids for either RNA trap or RBP (POI). Under the conditions tested, although a higher RNA trap amount increased the relative signal at the *lac*O foci both in the presence and absence of the ROI, the enrichment ratio between the experimental and background binding control groups was basically constant (Supplementary Figure S4A, B), suggesting the amounts of RNA trap used here are all redundant. We then optimized the amount of the POI (PCP-mCherry) for the assay and found that the 0.2 ng PCP-mCherry group performed as good as the 0.4 ng group, both showed higher enrichment of the RBP than the 0.8 ng group (Supplementary Figure S4C, D). This optimized POI amount was applied for the subsequent studies.

Moreover, we tried to enhance the trapping efficiency of the MCP RNA trap by doubling the ms2 RNA binding unit, and the RNA trap with two tandem ms2 binding units (2MCP RNA trap) showed a slight improvement of the POI enrichment over the original MCP RNA trap (Supplementary Figure S5). We also tested mScarlet-I, a brighter fluorescent protein than mCherry, to label the test protein, which worked as good as the mCherry for the assay, but not showing enhancement of the measurement or sensitivity as both the signal and background readout were raised (Supplementary Figure S6).

Different types of RNAs are transcribed, processed, modified and located differently in cells, which may affect the measurement of rF3H assay. Therefore, we compared two RNA transcription cassettes with different properties. In the CMV cassette, a CMV promoter (transcribed with RNA polymerase II) together with the SV40 polyadenylation (poly(A)) signal was applied for target RNA transcription; and for the U6 cassette, the RNA was transcribed via a U6 promoter (transcribed with RNA polymerase III), which does not cause additional poly(A) modification on the transcribed RNAs (Supplementary Figure S7A). We observed that the RNA generated from the U6 cassette resulted in a better recruitment of the RBP to *lac*O foci than RNAs produced by the CMV cassette (Supplementary Figure S7B, C). After obtaining the total cellular RNA and nuclear RNA from CMV or U6 cassette transfected cells, we measured the amount of the test RNA in these two components by qPCR respectively. We found that while the total RNA produced by CMV and U6 cassettes were almost the same (Supplementary Figure S7D), the U6 products showed higher nuclear retention (Supplementary Figure S7E), which may explain the different performances of the two cassettes for the assay.

### Visualization of mRNA–protein interactions with rF3H

**Figure 2.**
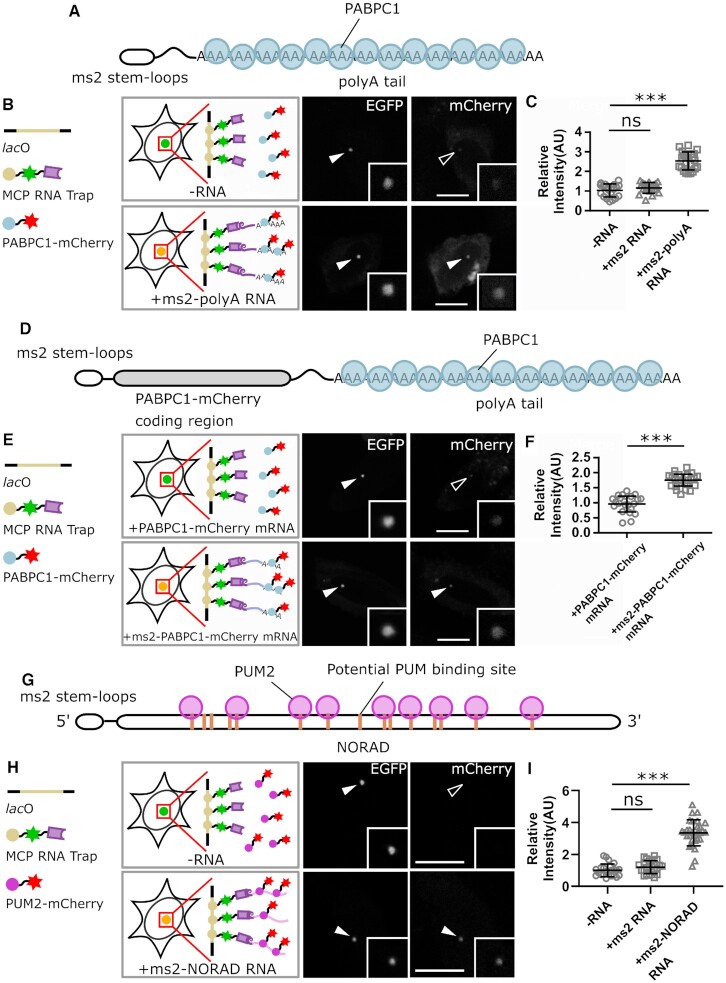
Detection of interactions between proteins and different types of RNAs by rF3H assay. (**A**–**C**) Interaction between protein and an mRNA mimic is detected by rF3H. The ms2 stem–loop RNA is capped at the 5-prime and polyadenylated at the 3-prime to mimic mRNA, and its interaction with the PABPC1 protein is illustrated in A. Representative images of the mCherry labeled PABPC1 protein enrichment at the lacO array in the presence and absence of ms2 mRNA mimic are shown in B, and complete images with controls are shown in Supplementary Figure S6. Quantification results revealed that the polyadenylated ms2 mRNA mimics largely enhanced enrichment of PABPC1 at the lacO site in comparison to the two controls (in C). Data are presented as mean ± S.D., for -RNA, *n* = 27; +ms2 RNA, *n* = 28; +ms2-polyA RNA, *n* = 27; *** *P* < 0.001. (**D**–**F**) Detection of interactions between protein coding mRNA and PABPC1. The structure of PABPC1-mCherry mRNA, which is labeled with ms2 stem–loops at the 5′ end, are shown in D. Enrichment of mCherry labeled PABPC1 protein at the lacO site, which is recruited by ms2 tagged PABPC1-mCherry mRNA, are detected (in E). In F, Quantification results showed that interacting with mRNA leads to an about 2 times enrichment of PABPC1 at the lacO spot, which is significantly different in comparison to the control group. Data are presented as mean ± S.D., for control and ms2 labeled mRNA, *n* = 26 and 27, respectively. *** *P* < 0.001. (**G**–**I**) Analysis of the interaction between NORAD ncRNA and PUM2 by rF3H assay. The NORAD ncRNA, which is a large non-coding RNA, contains more than 10 potential PUM protein binding sites (in G). The mCherry tagged PUM3 protein is recruited to the lacO array by the ms2 tagged NORAD RNA (in H). Quantification of the relative fluorescence at lacO spots showed a significant enrichment of the PABPC1 protein by interacting with the NORAD RNA (in I). Data are presented as mean ± S.D., for control, +ms2 RNA and NORAD RNA groups, *n* = 25, 25 and 27, respectively. ns: *P* > 0.05, *** *P* < 0.001. Scale bars represent 10 μm.

The mRNA is a large category of RNAs coding for proteins, and the synthesis, processing and ribosomal translation of mRNAs require interacting and formation of complexes with multiple proteins (23,24). For example, the poly(A) binding family proteins (PABPs) recognize poly(A) sequences at the 3′ end of the mRNA, and binding of PABPs to mRNAs facilitates mRNA translation and regulates mRNA stability (25,26). To test the feasibility of the rF3H assay on mRNA–protein interactions, we checked the interaction between a polyadenylated mRNA mimic and PABP1. The mRNA mimic, containing 2 times ms2 stem–loop structures, was transcribed from a CMV cassette and, therefore, modified with 5′ cap and 3′ poly(A) tail (Figure 2A). The mRNA mimic was trapped at the *lac* operator array by the MCP RNA trap, and recruitment of the mCherry tagged PABPC1 to the *lac*O array was observed in the presence of ms2 mRNA mimic (Figure 2B, Supplementary Figure S8A), indicating a physical interaction between the mRNA mimic and PABP1. Image quantification clearly showed that the enrichment of PABP1 at the *lac*O array doubled in the presence of the mRNA mimic, which is significantly different in comparison to the control groups (Figure 2C). In addition, we tested the interaction between PABPC1-mCherry mRNA and its products PABPC1-mCherry protein (Figure 2D). Anchored to the *lac*O array via the fused ms2 stem–loops, the mRNA successfully recruited PABPC1-mCherry protein to the *lac*O spot (Figure 2E, F, Supplementary Figure S8B), confirming the interaction between protein coding mRNA and PABPC1 protein.

### Interaction determination between non-coding RNA and protein

Long non-coding RNAs (lncRNAs) are non-coding RNAs with the length exceeding 200 nucleotides. Although thousands of lncRNAs are transcribed in the human genome, for most of them the functions are not known. The non-coding RNA activated by DNA damage (NORAD) is one conserved lncRNA transcribed in multiple species and is critical for the maintenance of genome stability (27,28). While NORAD depletion leads to premature aging and genome instability, the molecular mechanisms behind these phenotypes remain elusive. Sequence analysis indicated multiple PUMILIO family protein binding sites (with UGUANAAUA consensus sequence) in NORAD (Figure 2G). Therefore, we tested the interaction between the NORAD and PUMILIO 2 (PUM2) protein. We constructed a ms2 tagged NORAD and mCherry tagged PUM2 and measured their interaction with the rF3H assay. The co-localization of PUM2 and RNA trap was observed specifically when the NORAD RNA was transcribed (Figure 2H, Supplementary Figure S8C), and the quantification of mCherry-PUM2 showed a significant enrichment of the PUM2 protein at the *lacO* array recruited by NORAD RNA (Figure 2I), confirming the interaction between NORAD and PUM2 protein (29,30).

### Characterization of the interaction between EZH2 and HOTAIR

**Figure 3.**
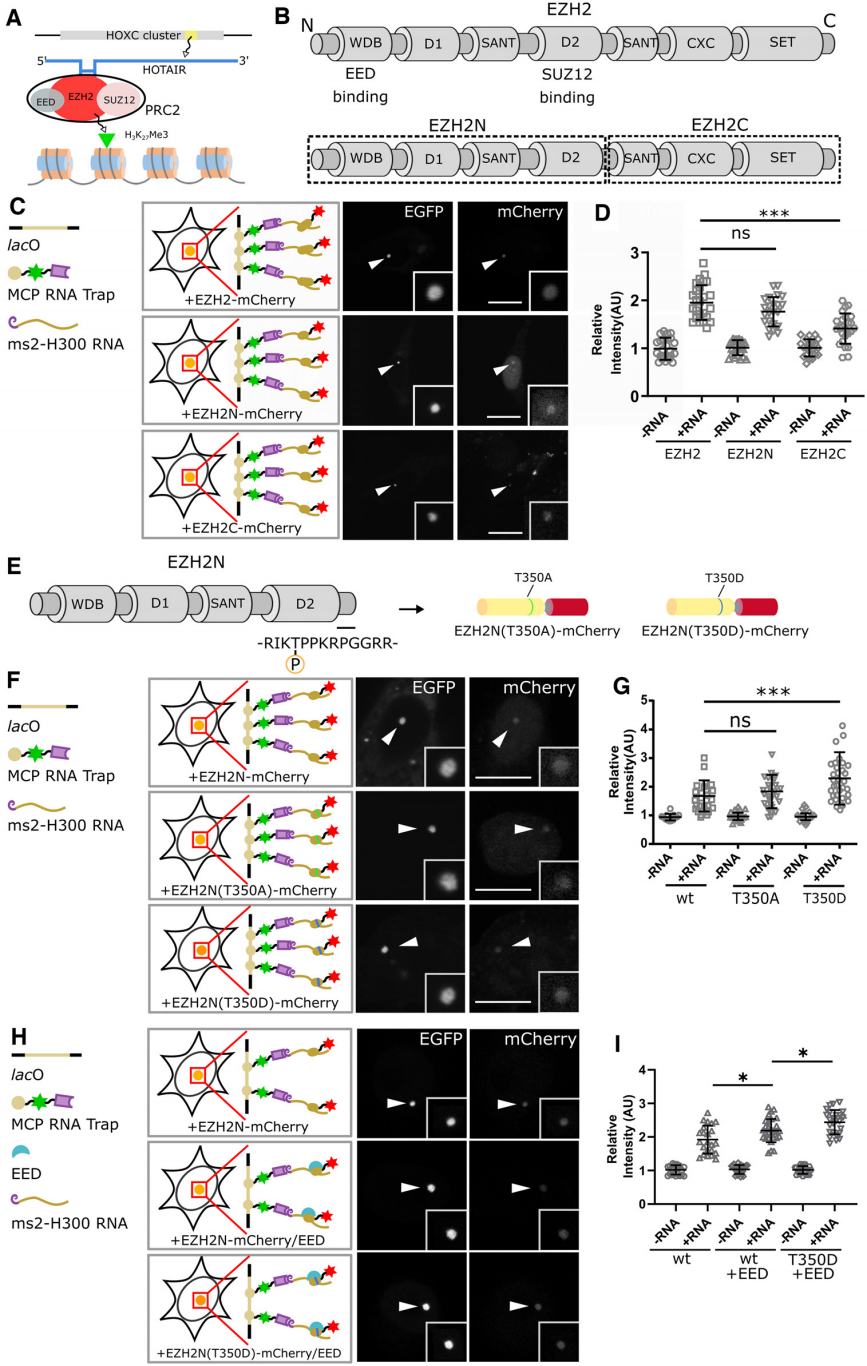
Characterization of the ncRNA binding property of EZH2 protein. (**A**) The HOTAIR ncRNA, which is transcribed from the HoxC cluster, interacts with the PRC2 complex and thus involves in regulation of tri-methylation on the 27th lysine of histone H3. (**B**) EZH2 protein was divided into two parts, and the N-terminal part of EZH2 (EZH2N), which contains four domains, was proposed to be the major part for ncRNA binding. (**C**) rF3H image examples showing the binding of full-length EZH2, EZH2N and EZH2C to HOTAIR H300 RNA. Both the full protein and the fragments showed RNA binding activities, and protein enrichments at the lacO spot are marked by arrowheads. (**D**) Image quantification demonstrated that the H300 RNA can lead to significant recruitments of both full- length and N-terminal EZH2 at the lacO array, with a relative weak recruitment of the EZH2C fragment. The N-terminal part of EZH2 harbors the most H300 binding ability compared with the full-length protein (see complete images with all controls in Supplementary Figure S9A). Data are presented as mean ± S.D., for the groups shown from left to right, *n* = 25, 27, 24, 22, 24, 26, respectively. *** *P* < 0.001. (**E**–**G**) Phosphorylation states affect the RNA binding ability of EZH2N protein. The threonine at the 350th position could be post-transcriptionally modified by phosphorylation, and two EZH2N mutants, T350A and T350D, were constructed by replacing the 350th threonine with alanine or aspartic acid to simulate the unphosphorylated or the phosphorylated states (in E). In (F), the recruitment of the mCherry labeled EZH2N, EZH2N (T350A) and EZH2N (T350D) protein at the lacO array by ms2 tagged H300 RNA are shown (see complete images with all controls in Supplementary Figure S9B). Quantitative assay in G indicated that the EZH2N (T350A) protein has a similar binding ability as the wild-type protein, while the T350D mutation showed a stronger binding activity to H300 RNA. Data are presented as mean ± S.D; for the groups shown from left to right, *n* = 24, 26, 25, 26, 23, 24, respectively. *** *P* < 0.001. (**H**, **I**) EED promotes EZH2N-HOTAIR interaction, which is further enhanced by phosphorylation. H shows the recruitment of EZH2N, EZH2N/EED complex and EZH2N(T350D)/EED complex at the lacO array by ms2 tagged H300 RNA (see complete images with all controls in Supplementary Figure S9C). Data are presented as mean ± S.D; for the groups shown from left to right, *n* = 26, 27, 26, 29, 24, 28, respectively. * *P* < 0.05. For all the images, scale bars stand for 10 μm.

Non-coding RNAs could also act as epigenetic regulators for modulation of gene expression. The HOX transcript antisense intergenic RNA (HOTAIR) is an ncRNA transcribed from the Homeobox C (HoxC) cluster (31). This ncRNA forms RNP complexes with certain epigenetic modulators, like the PRC2 complex, to regulate histone methylation and gene expression (Figure 3A). One of the major components of PRC2, the enhancer of zeste homolog 2 (EZH2) protein, has been shown to interact with the first 300 nucleotides of HOTAIR (32,33), and its N-terminal part was considered to play a critical role in RNA binding (34–37) (Figure 3B). To validate the interaction between HOTAIR and EZH2 (full-length, N- and C-terminal parts), we constructed mCherry labeled full-length as well as N-terminus (EZH2N) and C-terminus (EZH2C) of EZH2 and determined their interactions with ms2 tagged HOTAIR 1–300 nt (H300). Our results showed that both the EZH2N and EZH2C are recruited to the *lac*O spot by the H300 RNA fragment (Figure 3C, Supplementary Figure S9A) albeit EZH2C at a lower extent. EZH2N showed a comparable binding to H300 RNA as the full-length EZH2 (Figure 3D), which indicates that EZH2N preserves most RNA binding ability of the full-length EZH2 and likely plays a major role in HOTAIR binding, while EZH2C also kept some RNA binding ability, both results were similar to the discoveries in a previous study (36).

Post-transcriptional modifications, especially phosphorylation play crucial roles in regulation of protein activities. Prior studies showed that several residues of EZH2 could be modified by phosphorylation (38,39), and the phosphorylation of the threonine at the 350th position was proposed to affect its ncRNA binding activity (34). To find out whether this phosphorylation influences the interaction between EZH2N and HOTAIR H300 RNA, we constructed a phosphorylation mimic of EZH2N by substitution of threonine at 350th position with aspartic acid (T350D) as well as an alanine mutant (T350A) for non-phosphorylation mimic (Figure 3E). Both the phosphorylation and non-phosphorylation mimics bound HOTAIR H300 at the *lac*O site (Figure 3F, Supplementary Figure S9B), and the phosphorylation mimic T350D showed a higher binding ability to HOTAIR H300 (Figure 3G), providing the possibility that T350 phosphorylation may regulate RNA binding ability of EZH2N. As the RNA binding sites in EZH2N were considered distantly from this position (36), we believed the regulation was performed by some indirect effects such as conformational change caused by phosphorylation. EED, another major component of the PRC2 complex, was considered to facilitate EZH2-HOTAIR formation (32). Our approach demonstrated that EED is able to contribute to the binding between EZH2N and HOTAIR H300 RNA, and the phosphorylation of EZH2N T350 can further enhance this interaction (Figure 3H, I, Supplementary Figure S9C).

**Figure 4.**
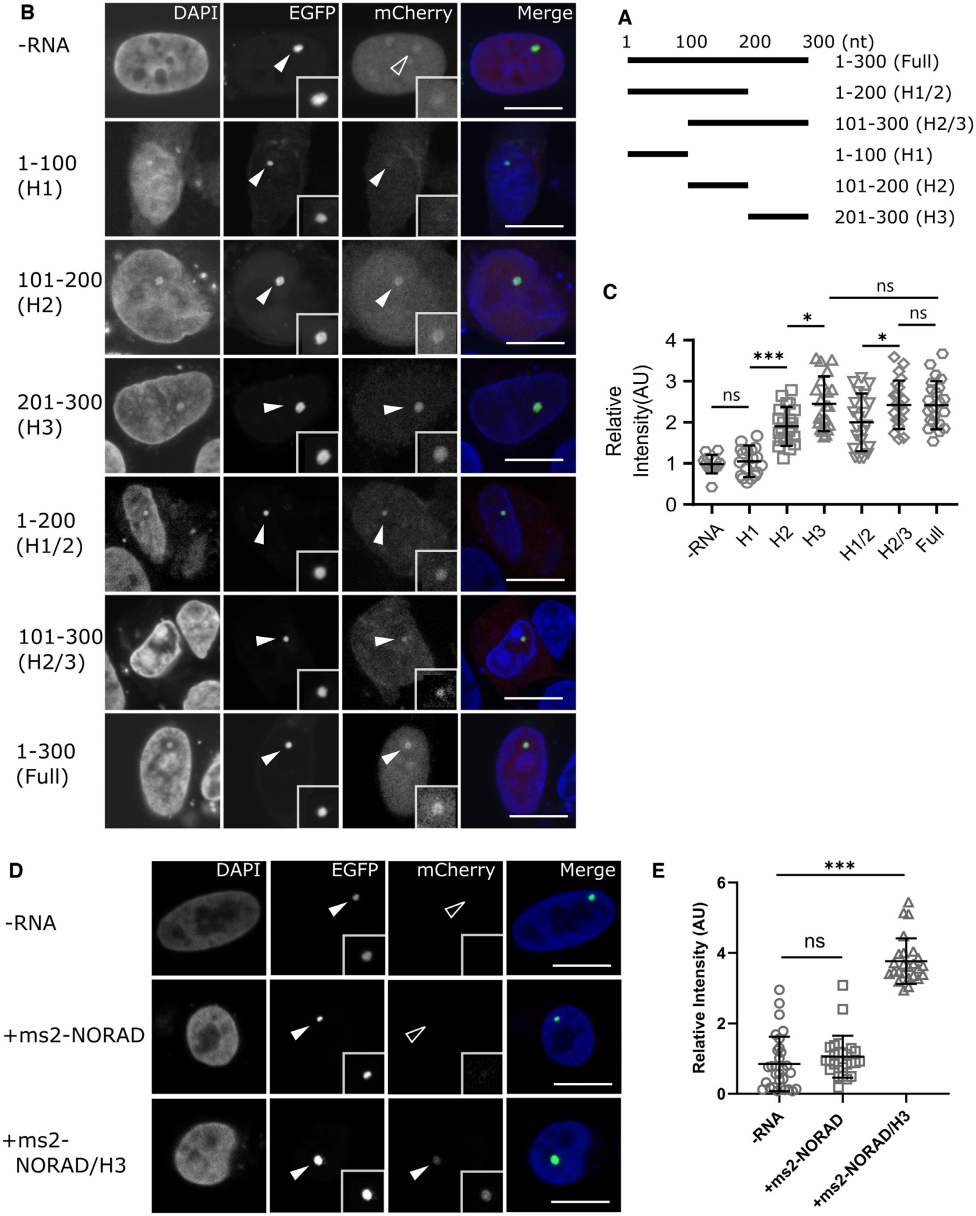
Characterization of HOTAIR H300 fragments that binds to EZH2. (**A**) Five H300 sub-fragments, which include 1–100 nucleotides (H1), 101–200 nucleotides (H2), 201–300 nucleotides (H3), 1–200 nucleotides (H1/2) and 101–300 nucleotides (H2/3), were constructed for EZH2 binding tests. (**B**) Representative images of mCherry tagged EZH2 protein recruitment at the lacO array by different HOTAIR H300 sub-fragments. (**C**) Quantitative image analyses showed that all fragments except H1 bind to EZH2 protein, and fragments containing 201–300 part showed a similar binding ability with the full H300 fragment, indicating the 201–300 is the major EZH2 binding part. Data are presented as mean ± S.D., for the groups shown from left to right, n = 22, 24, 23, 28, 27, 26, 27, respectively. ns *P* > 0.05, * *P* < 0.05, *** *P* < 0.001. (**D**) Images revealed the localization of mCherry tagged EZH2 protein at the lacO site by ms2 tagged NORAD/H201-300 chimeric RNA. (**E**) Quantification demonstrates that EZH2 can bind NORAD/H201-300 RNA while this was not the case with the original NORAD RNA. Data are presented as mean ± S.D., from left to right, *n* = 28, 28, 27, respectively. ns: *P* > 0.05, *** *P* < 0.001. For all the images, merge channels were enhanced for clarity purposes and scale bars stand for 10 μm.

To narrow down the HOTAIR sequences responsible for EZH2 binding, we divided the HOTAIR H300 into smaller fragments and identify the interactions between them and EZH2 protein. As shown in Figure 4A–C, three basic RNA fragments (HOTAIR 1–100 nt, HOTAIR 101–200 nt and HOTAIR 201–300 nt) and two combinations (HOTAIR 1–200 nt and HOTAIR 101–300 nt) were tagged with ms2 loops and checked for their interactions with EZH2. All the truncations except the HOTAIR 1–100 nt one showed recruitment of EZH2-mCherry, revealing that the first 100 nucleotides of the HOTAIR are dispensable for EZH2 binding. Quantitative analyses further showed that the fragments containing 200–300 nt part had a higher enrichment of EZH2 than others, which indicates that the 200–300 nt part of HOTAIR is the major EZH2 binding region, consistent with a previous study (32). Furthermore, a chimeric NORAD/HOTAIR 201–300 RNA acquired the ability to bind EZH2, which further confirmed the result (Figure 4D, E).

The research between three different RNA–protein pairs showed the reliable application of this rF3H assay in RNA–protein interaction study. In comparison to traditional biochemical assays, this imaging-based method is more flexible, less labor intensive, quantitative and with high throughput. And more importantly, interaction information is obtained in individual cells, instead of the average of a whole-cell population as in traditional biochemical assays. Besides, RNA protein interaction can be analyzed in living cells under different culture conditions, therefore, characterization of the RNA–protein dynamics can be studied and issues like heterogeneity in a cell population or RNP formation/disassembly can possibly be addressed with this assay, providing deeper insights on RNA–protein interaction within the cellular context.

### Detection of RNA–protein interactions at multiple cellular structures

**Figure 5.**
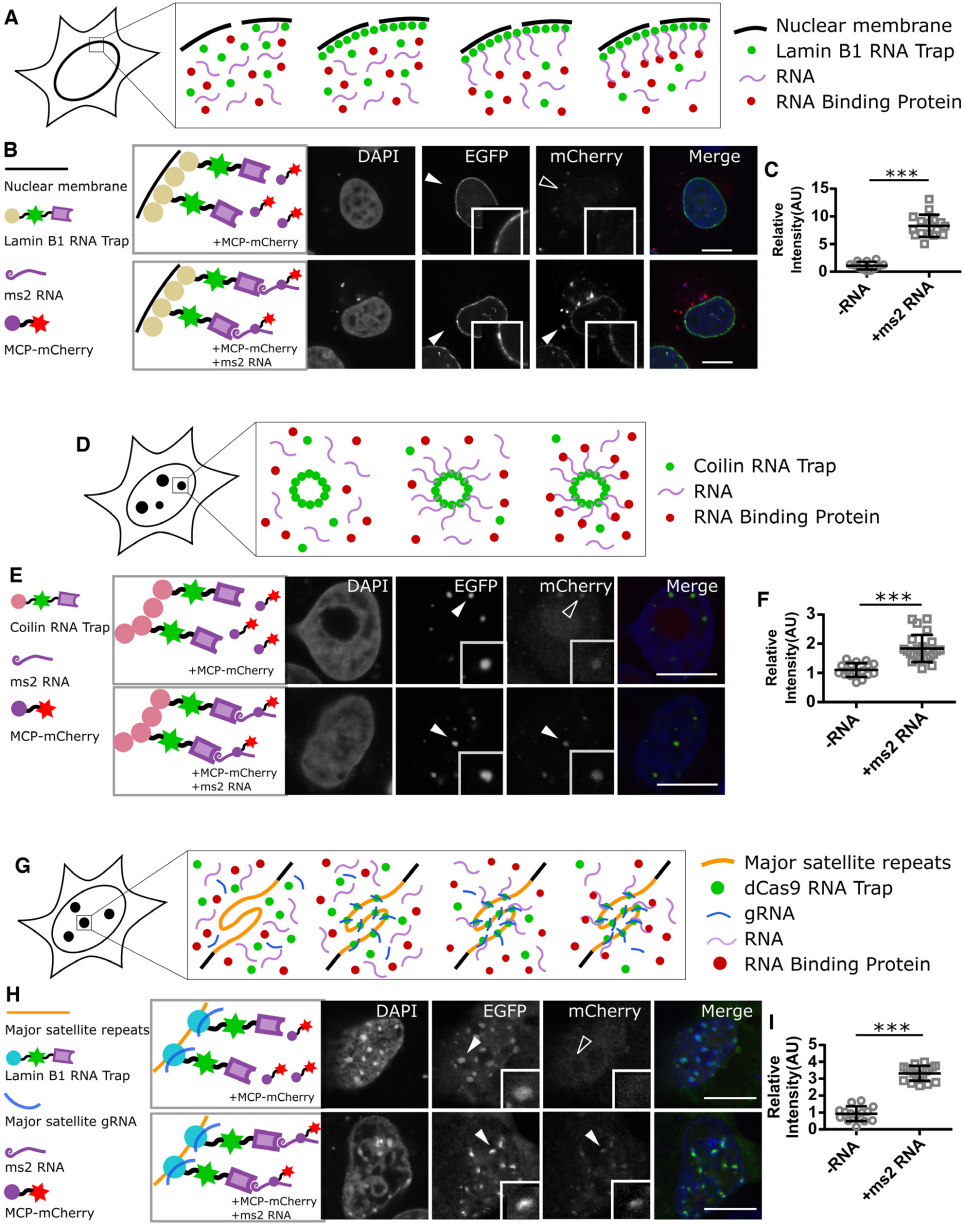
Development of multiple anchor sites for rF3H assay. (**A**) Schematic diagram of rF3H on the nuclear envelope. A Lamin B1 containing RNA trap, which anchors to the inner nuclear membrane, is able to recruit test RNAs, thus allowing for measurement of RNA–protein interaction at the nuclear envelope. (**B**) Detection of mCherry labeled MCP protein (mCherry channel) interacting with ms2 RNA at the inner nuclear membrane in HeLa cells (Scale bar stands for 10 μm). (**C**) Quantitative analysis showed that the enrichment of the MCP protein at the inner nuclear membrane is enhanced significantly by interacting with the anchored ms2 RNA. Data are presented as mean ± S.D., for the control and +ms2 RNA group, *n* = 21, 24, respectively. *** *P* < 0.001. (**D**) Schematic illustration of rF3H assay at Cajal nuclear bodies in HeLa cells. The test RNA molecules are trapped at the Cajal nuclear bodies by the Coilin RNA trap, and RBP would be recruited to the same cellular structure serving as indication for RNA–protein interaction. (**E**) The accumulation of mCherry labeled MCP proteins at the Cajal nuclear bodies without and with ms2 RNA (scale bar 10 μm). (**F**) Quantification of the MCP protein at the Cajal bodies showed a significant increase in the presence of ms2 RNA, clearly confirming the interaction between the RNA and protein. Data are presented as mean ± S.D., for the control and +ms2 RNA group, *n* = 22, 26, respectively. *** *P* < 0.001). (**G**) Chromatin structures are developed as anchor sites for rF3H assay in J1 cells. RNA traps containing deactivated Cas9 (dCas9) can bring the test RNA to chromocenters with guide RNAs targeting the major satellite repeats, so that the RBP is recruited to chromocenters by interacting with the trapped RNAs. (**H**) The accumulation of the mCherry tagged MCP protein at chromocenters in the presence and absence of ms2 RNA (scale bars equal to 10 μm). (**I**) Quantitative analyses showed an about three times higher enrichment of MCP proteins at chromocenters by interacting with ms2 RNA than the control without ms2 RNA, which is significantly different (data are presented as mean ± S.D., for the control and +ms2 RNA groups, *n* = 23, 26, respectively. *** *P* < 0.001).

The LacI fused RNA trap requires the special *lac*O array containing cell lines for anchoring of RNAs. To overcome this limitation, we developed a RNA trap that targets RNAs to the nuclear lamina by fusing the RNA binding unit to Lamin B1 protein, which is a major component of the nuclear lamina (Figure 5A) (40–42). The RNA molecules, in association with the RNA trap, were successfully anchored to the inner nuclear membrane (INM) in HeLa cells and the interactions between ms2 RNA and MCP protein could be detected on the nuclear envelope (Figure 5B, C), supporting an rF3H assay on natural subcellular structures.

Nuclear bodies are membrane-less organelles in the cell nucleus with multiple functions, including RNA processing (43–45). Next, we developed an RNA trap fused to Coilin protein, which is the major component of the Cajal nuclear bodies (46–49), to anchor the RNA of interest specifically there (Figure 5D). As expected, RNA traps were detected as fluorescent nuclear spots, and the interaction between ms2 RNA and MCP protein can also be visualized clearly on the Cajal nuclear bodies in HeLa cells (Figure 5E, F).

Catalytically deactivated Cas9 (dCas9) protein has been used for targeting and visualization of genomic loci (20,50,51). Taking the advantage of this versatile technique, we designed a RNA trap that applies dCas9 to anchor RNAs to genomic structures (Figure 5G). Guided by gRNAs targeting genomic major satellite repeats, this dCas9 fused RNA trap anchored ms2 RNA molecules to chromocenters in mouse embryonic stem cells (mESCs), being visualized as multiple nuclear spots (Figure 5H, I). Accordingly, the mCherry tagged MCP protein was recruited to chromocenters by the trapped ms2 RNAs, showing a successful detection of RNA–protein interactions with this dCas9 based RNA trap.

All together, RNA traps anchoring to these multiple subcellular structures expanded the applicability of the rF3H assay, suitable for characterization of the interaction between RNAs and proteins with different properties.

### Development of PUF and dCas13a mediated RNA trapping systems

**Figure 6.**
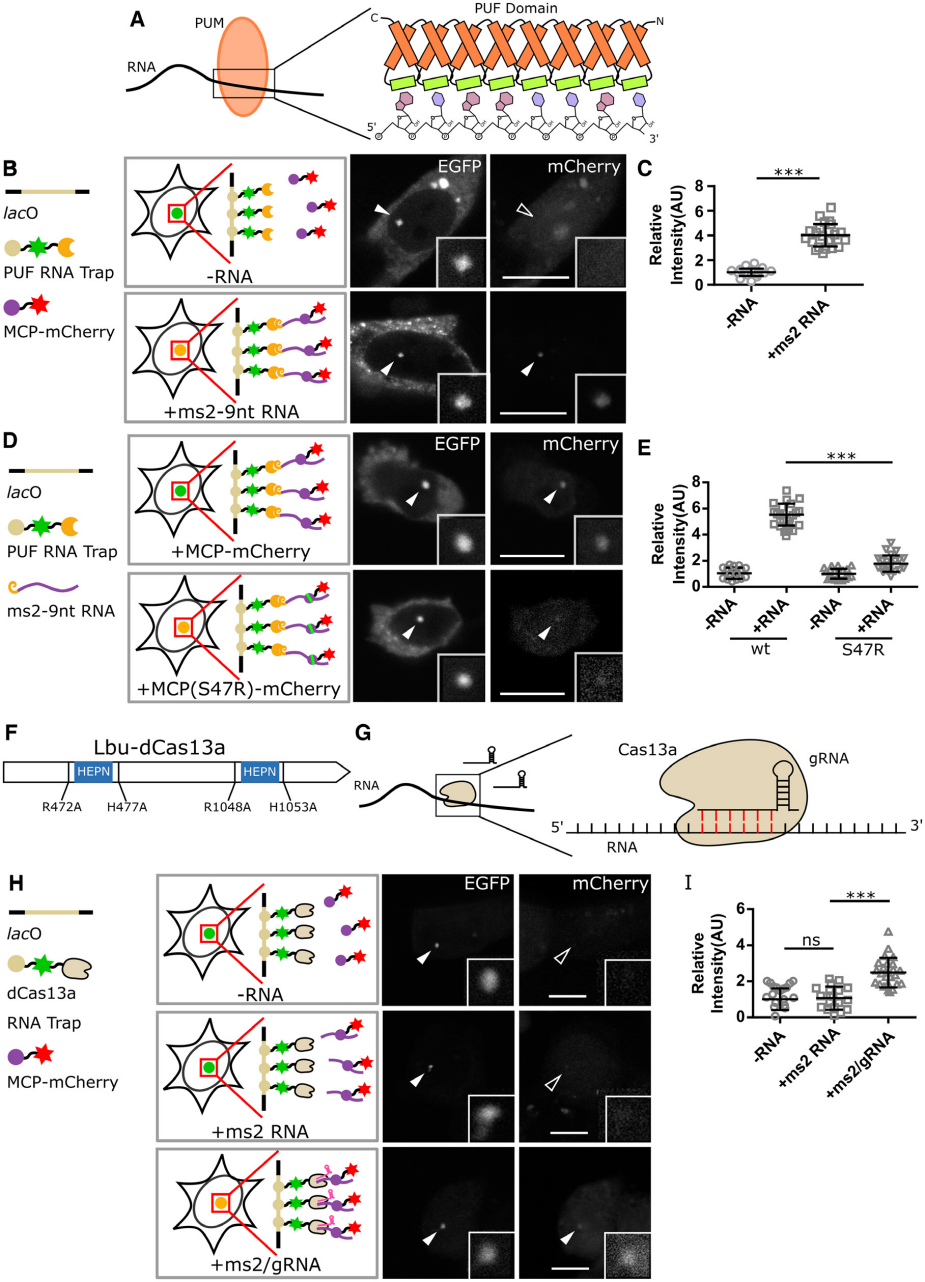
Development of programmable RNA traps that can recognize natural RNAs for rF3H assay. (**A**–**E**) PUF RNA traps are developed for RNA–protein interaction assays. The PUF domain of PUM proteins typically contains eight tri-helix repeats, each of which responds to the single nucleotide recognition, and can be engineered for binding of certain RNAs (in A). In B, the ms2 RNA containing a PUF recognizable 9-nt RNA sequence was tested and trapped at the *lac*O array by a PUF RNA trap (EGFP channel), and the enrichment of mCherry tagged MCP proteins at the *lac*O array can be observed. Image quantification demonstrated a significant enrichment of the MCP protein at the *lac*O site caused by ms2 RNA (in C, data are presented as mean ± S.D, *n* = 22, 26, *** *P* < 0.001). Based on the PUF RNA trap, rF3H images for comparison of the wild-type and mutant (S47R) MCP proteins recruitment at the *lac*O array are shown (in D), and quantitative analyses showed that the mutant MCP possesses a much weaker ms2 RNA binding ability than the wildtype, demonstrating sensitive detection of different binding affinities by rF3H assay (in E, data are presented as mean ± S.D., for the groups shown from left to right, *n* = 23, 26, 24, 28, respectively). (**F**–**I**) Development of rF3H assay with dCas13a RNA trap. For Lbu-Cas13a protein, while the nuclease activity can be eliminated by four mutations (R472A, H477A, R1048A, H1053A) in the two HEPN domains, its RNA targeting ability is reserved and this deactivated Cas13a can bind to RNAs in the presence of a gRNA (in F, G). In H, the ms2 RNA is targeted and trapped by the dCas13a RNA trap with a gRNA specifically recognizing the ms2 RNA (EGFP channel), recruitment of the mCherry labeled MCP protein at the *lac*O site could be detected only in the presence of both the ms2 RNA and gRNA, but not in control groups. Quantification results revealed a two-time higher enrichment of MCP protein than the control groups, confirming the performance of the cas13a RNA trap in rF3H assay (in I, data are presented as mean ± S.D., *n* = 21, 23, 26, *** *P* < 0.001). Scale bars stand for 10 μm.

Besides ms2-MCP RNA binding pair, RBPs which bind RNA in a sequence-specific manner with high binding ability could also be engineered for trapping of RNAs. As we have shown previously, the PUMILIO family proteins (PUMs) bind to RNAs containing the PUM binding sequences via the Pumilio and FBF homology (PUF) domain. The PUF domain typically consists of multiple tri-helix motifs, and the crystal structure showed that each of the tri-helix motifs recognizes and binds one nucleotide, which makes the PUF domain a suitable candidate for RNA binding engineering (Figure 6A) (52–54). Using a designed synthetic PUF domain that recognizes a nine-nucleotide sequence (UGUUGUAUA) (55), we constructed an RNA trap anchoring the corresponding target RNAs at the *lac*O spot. The ms2 RNAs tagged with the 9-nt PUF binding sequence were recruited and visualized by the GFP fused PUF RNA trap, and enrichment of the mCherry tagged MCP at the *lac*O array was detected by confocal imaging (Figure 6B, C, Supplementary Figure S10A). Although a nuclear localization signal was added to the PUF RNA trap fusion, part of the protein remains cytoplasmic. Nonetheless, enough protein is nuclear to score binding or lack thereof. Moreover, with this PUF RNA trap the reduced RNA binding ability of the S47R mutant MCP could also be detected (Figure 6D, E, Supplementary Figure S10B), which demonstrates the sensitive detection of RNA–protein interactions with this PUF based RNA trap.

The CRISPR/Cas system is a prokaryotic defense mechanism against foreign viral nucleic acids. Besides the Cas9 protein, which is a DNA nuclease, Cas proteins that cut RNAs have also been identified recently (56–58). Cas13a is one of such RNA-activated RNases which target and cut RNA molecules specifically under the control of small gRNAs (59,60). Similar to Cas9, catalytically deactivated Cas13a (dCas13a) has also been applied for RNA tracking and visualization in cells (61,62) (Figure 6G). Featured by flexible RNA targeting, we generated a *Leptotrichia buccalis*-sourced dCas13a (Lbu-dCas13a) derived RNA trap, together with the corresponding gRNA that recognizes a specific RNA sequence (GAU UCU AGA ACU AGU GGA UCC UAA GGU A) in the 5′ of ms2 RNAs. The dCas13a RNA trap showed successful trapping of target RNAs and detected RNA–protein interactions as shown by ms2-MCP interaction pair (Figure 6H, I, Supplementary Figure S10C), providing another potential tool for endogenous RNA capture.

In the beginning, our MCP RNA trap was designed for RNAs tagged with ms2 stem loops, now, the engineered PUF RNA trap, and also the dCas13a based RNA trap, have the potentials that broadly used for programmable targeting and binding of RNAs with ideally any sequences. These two types of RNA trapping systems offer valuable endogenous RNA targeting and trapping tools, allowing for flexible study of not only interactions but also the dynamics of endogenous RNAs and RNPs. Besides, multiple subcellular structures in both nuclei and cytosol were developed for RNA anchoring to fit different types of RNAs and protein. Furthermore, combining various subcellular localization components and programmable RNA catching tools, RNA trap could show the interaction between different RNA and proteins in suitable subcellular positions with immunofluorescence, which further expands the spectrum of its applications.

### Detection of RNA–protein binding in living cells

As RNA–protein interactions are usually dynamic under physiological conditions, detection of the interaction in living cells may offer insights on the dynamic regulation of RNPs. To extend the rF3H assay from fixed cells to living cells, we tested the well characterized pp7-PCP interaction under live conditions. As shown in Supplementary Figure S11, enrichment of MCP RNA traps, as well as mCherry fused PCP at the *lac*O site in the nucleus could be observed in living cells. As homeostasis of RNP complexes is tightly controlled to ensure their proper functions in cells, the dynamics of RNA–protein interactions can provide more insights into their functions. Our RNA trap system and the rF3H assay allows us to study these dynamical RNA–protein binding processes under physiological conditions. In combination with other technologies such as Fluorescence Recovery After Photobleaching (FRAP), precise measurements of RNA–protein binding kinetics may offer quantitative data on these dynamic processes.

## DATA AVAILABILITY

All data could be found in paper or supplementary data. Additional data could be requested from the corresponding authors.

## Supplementary Material

gkab614_Supplemental_File
