# Supplementary material for: Visualization and characterization of RNA–protein interactions in living cells
Source: Nucleic Acids Res. 2021 Jul 27;49(18):e107. doi: 10.1093/nar/gkab614 (PMC8501972; doi:10.1093/nar/gkab614)
Supplement: gkab614_Supplemental_File [file gkab614_supplemental_file.pdf]

Supplementary Material

Visualization and characterization of RNA-protein interactions in living cells

Ningjun Duan[1,4,*], Maria Arroyo[3], Wen Deng[1,2], M. Cristina Cardoso[3] and Heinrich Leonhardt[1,*]

1 Department of Biology II, Ludwig Maximilians University Munich, Großhaderner Str. 2, 82152 Planegg--Martinsried, Germany

2 College of Veterinary Medicine, Northwest A&F University, Yangling, Shaanxi, 712100, China

3 Department of Biology, Technical University of Darmstadt, Schnittspahnstr. 10, 64287 Darmstadt, Germany

4 Department of Oncology, The First Affiliated Hospital of Nanjing Medical University, Nanjing 210029, China

*** Corresponding author: Ningjun_official@outlook.com, h.leonhardt@lmu.de**

Supplementary Table 1

Source and catalog information of fragments used in plasmid construction.

Fragment	Full name	Catalog	Source/Reference
EGFP	pEGFP-N1	6085-1<br>pc0713	Clontech
EYFP	pEYFP-N1	6006-1<br>pc0715	Clontech
EED	pEGFP-N1-EED	pc3476	Kathrin Plath and (1)
LacI	pGFPBinder-LacI	pc1398	(2)
MCP	pGEX2TMS2	pc1827	Peter Becker and (3)
dCas9	pCAG-dCas9	pc2946	(4)
Lamin B1	pEGFP-LaminB1	pc1084	(5)
Coilin	pEcoilin-CFP	pc1298	(6)
PABPC1	pCI-MS2V5-PABPC1	65807<br>pc4653	Addgene and (7)
PUM2	pFRT/FLAG/HA-DEST PUM2	40292<br>pc4654	Addgene and (8)
NORAD	pcDNA3.1-NORAD	120383<br>pc4655	Addgene and (9)
Lbu-dCas13a	pDuBir-Lbu-dCas13a-avitag	100817<br>pc4656	Addgene and (10)
HOTAIR	pLZRS-HOTAIR	26110<br>pc4657	Addgene and (11)

Supplementary Figures

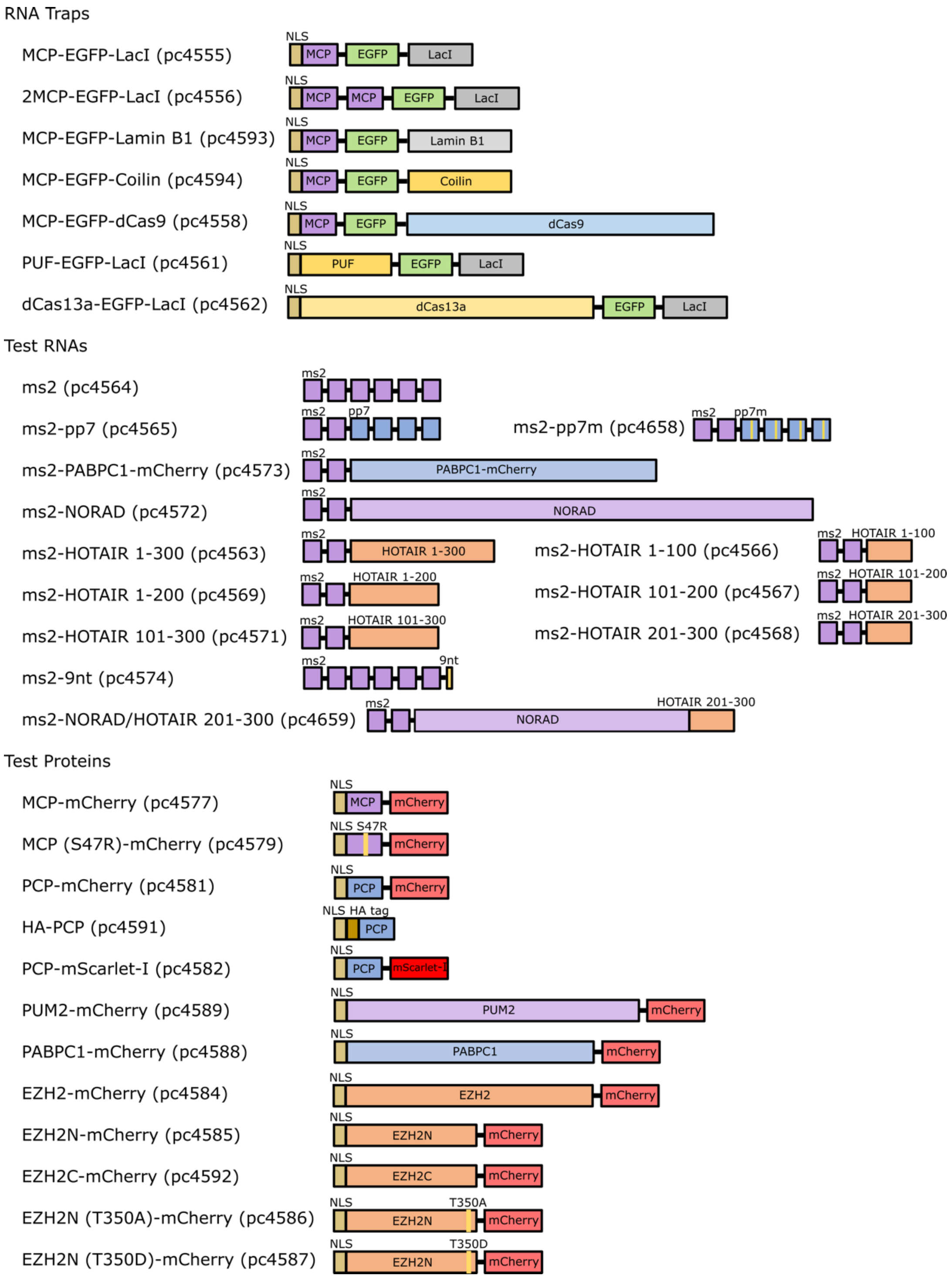

Supplementary Figure 1

Summary of the main functional elements of the constructs for the RNA trap, RNA and proteins.

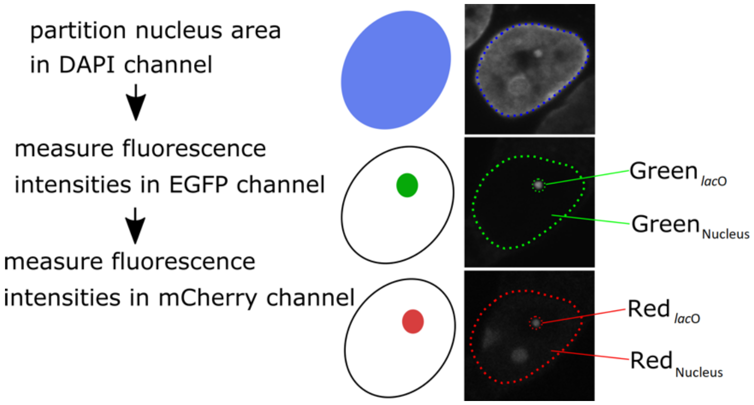

Supplementary Figure 2

Image analysis pipeline to obtain the fluorescence intensities in cells. The DAPI channel is used to segment the area of the nucleus, then the mean fluorescence intensities of the *lac*O spots and entire nucleus were measured in the EGFP channel and the mCherry channel, respectively.

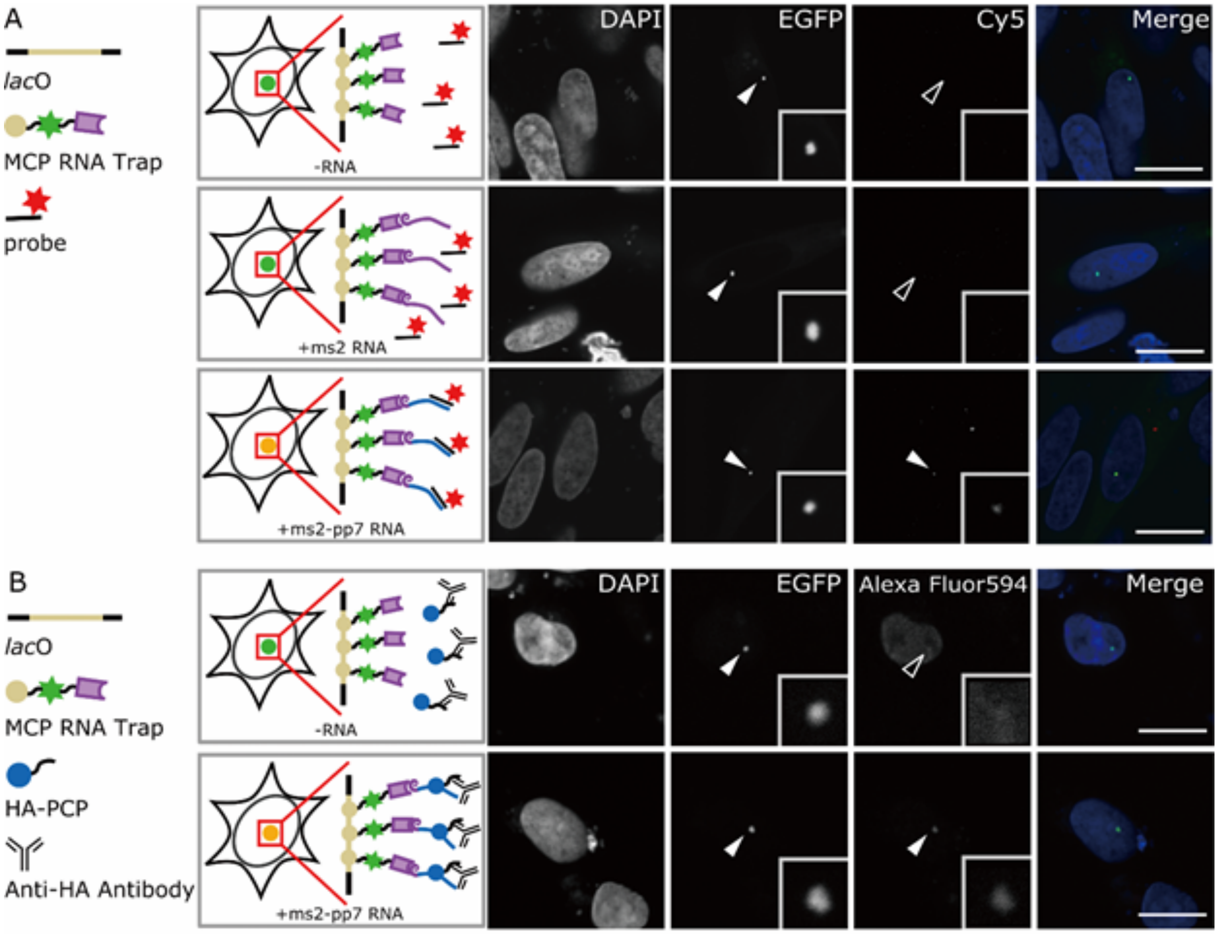

Supplementary Figure 3

(A) Detection of RNA trapped at *lac*O sites by FISH. Cy5 labeled anti-pp7 oligonucleotide probes were applied to detect pp7 RNA, and pp7 enrichment was observed specifically at the *lac*O sites, which confirms the successful trapping of ROI at the *lac*O site by the MCP RNA trap. **(B)** rF3H detection of pp7-PCP interaction by immunofluorescence. HA tagged POI (HA-PCP) was used instead of the mCherry-PCP for the assay, and the enrichment of POI was detected by antibody binding to HA tag (Alexa Fluor 594). For all the images, *lac*O sites are marked with arrowheads (filled arrowheads: signal enrichment; open arrowheads: no signal enrichment). Scale bars stand for 10 µm.

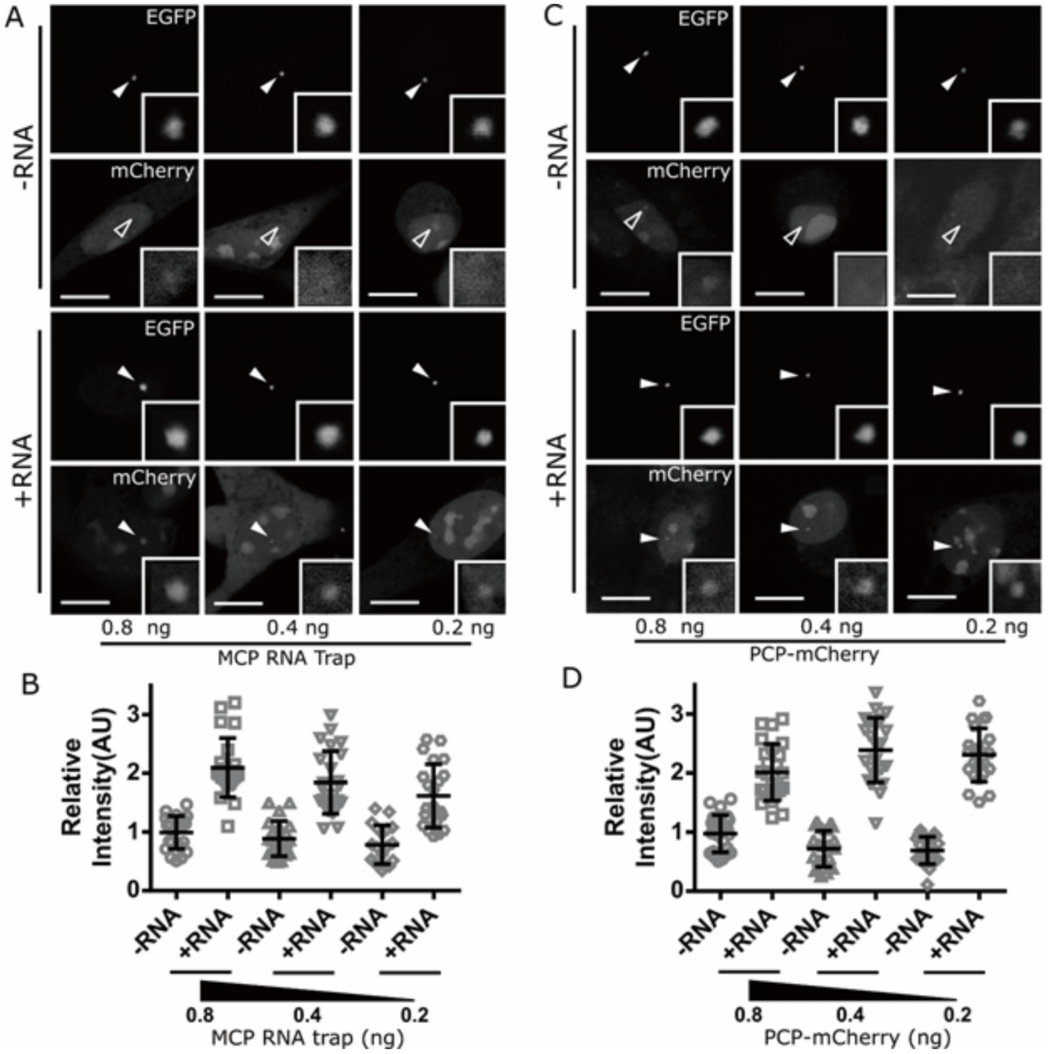

Supplementary Figure 4

The amount of RNA trap or test protein influences interaction assay **(A)** rF3H images with different amounts of RNA trap used for the assay (0.8 ng, 0.4 ng, 0.2 ng). rF3H performed without RNA (-RNA) serves as control for signal from background binding. **(B)** Image quantification shows an enhanced relative signal at the *lac*O spot along with the increase of RNA trap used, but also with increased background binding in the absence of RNA, resulting similar signal to background binding ratio for all the three groups (data are presented as mean ± S.D., for the groups shown from left to right, *n* = 25, 30, 28, 28, 25, 28, respectively). **(C)** rF3H images with different amounts of PCP protein (0.8 ng, 0.4 ng, 0.2 ng). rF3H performed without RNA (-RNA) serves as control for signal from background binding. **(D)** Image quantification showed that 0.2 ng and 0.4 ng groups both resulted in higher relative signals and lower background at the *lac*O spot than the 0.8 ng group (data are presented as mean ± S.D., for the groups shown from left to right, *n* = 27, 28, 26, 28, 26, 27). For all images, scale bars represent 10 µm.

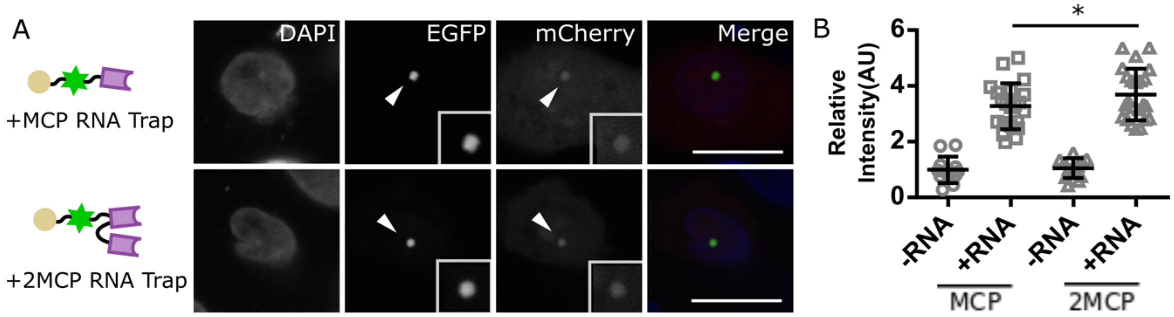

Supplementary Figure 5

Enhancement of rF3H performance by doubling the RNA trapping unit in the RNA trap. (**A**) Representative rF3H images from RNA traps with single (MCP RNA trap) and double (2MCP RNA trap) RNA binding units for detecting the interaction between pp7 and PCP protein (scale bars represent 10 μm). (**B**) Quantification of relative PCP signals at *lac*O spots showed an increased PCP recruitment by the 2MCP RNA trap (2MCP). Data are presented as mean ± S.D., for the groups shown from left to right, n = 22, 27, 23, 29, * $P < 0.05$.

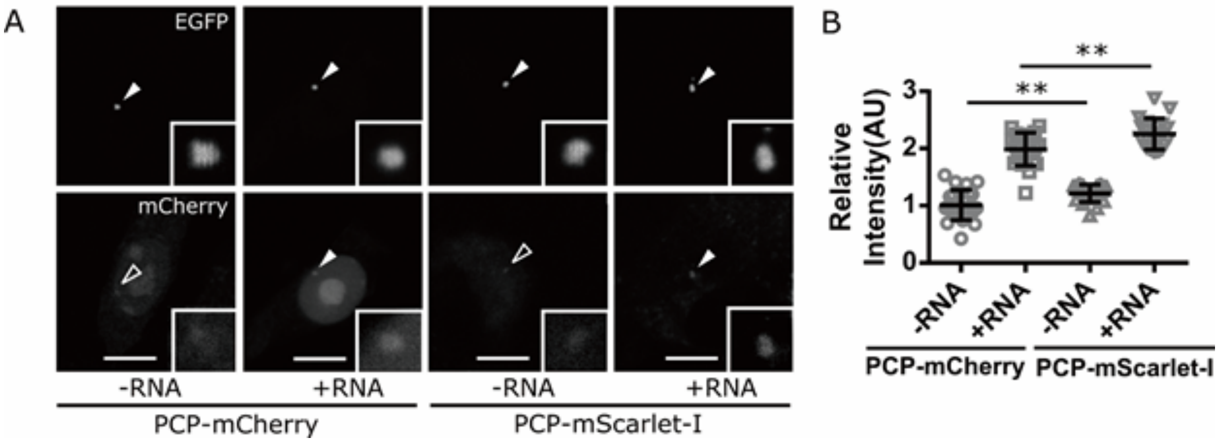

Supplementary Figure 6

Tests of different fluorescence protein tags for rF3H assay. **(A)** rF3H images of pp7-PCP interaction with mCherry or mScarlet-I tagged PCP proteins. Enrichment of PCP tagged by both fluorescent proteins could be observed (filled arrowheads), but not in controls without pp7 RNA (empty arrowheads). Scales bars represent 10 µm. **(B)** Quantitative analyses of the fluorescence signals detected for PCP tagged with mCherry and mScarlet-I. Image quantification showed that mScarlet-I, which is brighter than mCherry, resulted higher relative signals at the *lac*O spot both with and without binding RNAs, but the readout ratio (ratio between +RNA and -RNA) is almost identical to the mCherry tagged group, indicating fluorescent protein itself does not affect the rF3H results (data are presented as mean ± S.D., for the groups shown, from left to right, n = 28, 29, 27, 28. ** $P < 0.01$).

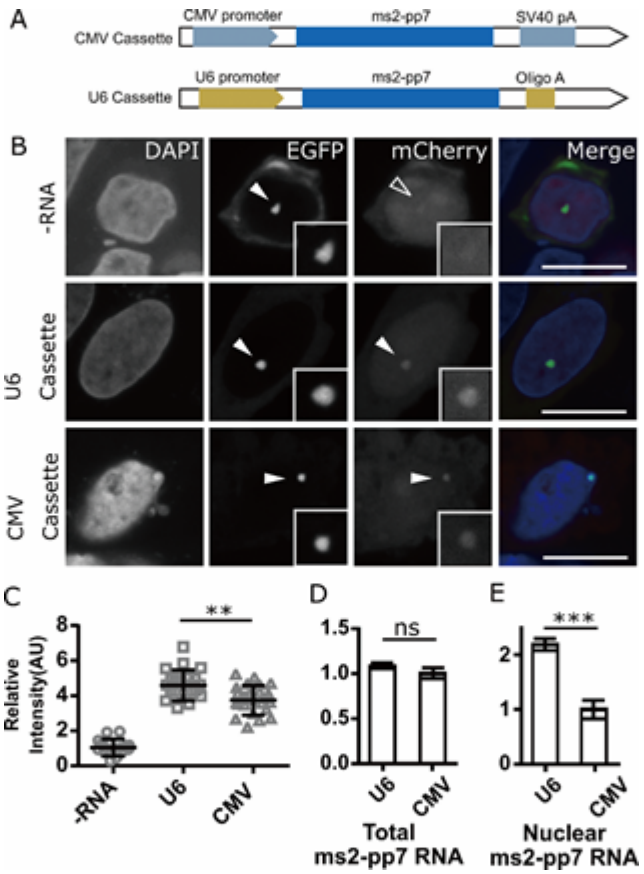

Supplementary Figure 7

Comparison of the RNA transcription cassettes for rF3H assay. **(A)** The structure of CMV and U6 cassettes for RNA transcription. In the CMV cassette, the ms2-pp7 RNA transcription is under the control of a CMV promoter and poly(A) sequences are added to the 3' mediated by an SV40 poly(A) signal sequence, while in the U6 cassette, U6 promoter and Oligo(A) sequence regulate the expression of the ms2-pp7 RNA. **(B)** Images of mCherry tagged PCP protein recruitment at the *lac*O array by ms2-pp7 RNA that was produced from U6 or CMV cassettes. Scale bars stand for 10 µm. **(C)** Quantification of the PCP protein signal at *lac*O spots showed that the RNA products from the U6 cassette led to a higher enrichment of the PCP protein than that from the CMV cassette products. Data are presented as mean ± S.D., for control, U6 and CMV groups, n = 23, 27 and 26, respectively. ** $P < 0.01$. **(D** and **E)** Comparison of test RNA generated by CMV and U6 promoter in different cellular parts by qPCR. The amounts of ms2-pp7 RNA generated from the CMV and U6 cassettes were almost the same, as quantified in **D**, but the U6 cassette products showed a one-fold higher concentration in the nucleus than the CMV products (in **E**), which may explain the better rF3H performance with RNAs from the U6 cassette. (Each group

contains three biological repeats, and the standard deviation was shown as an error bar. ns: not significant, *** $P < 0.001$)

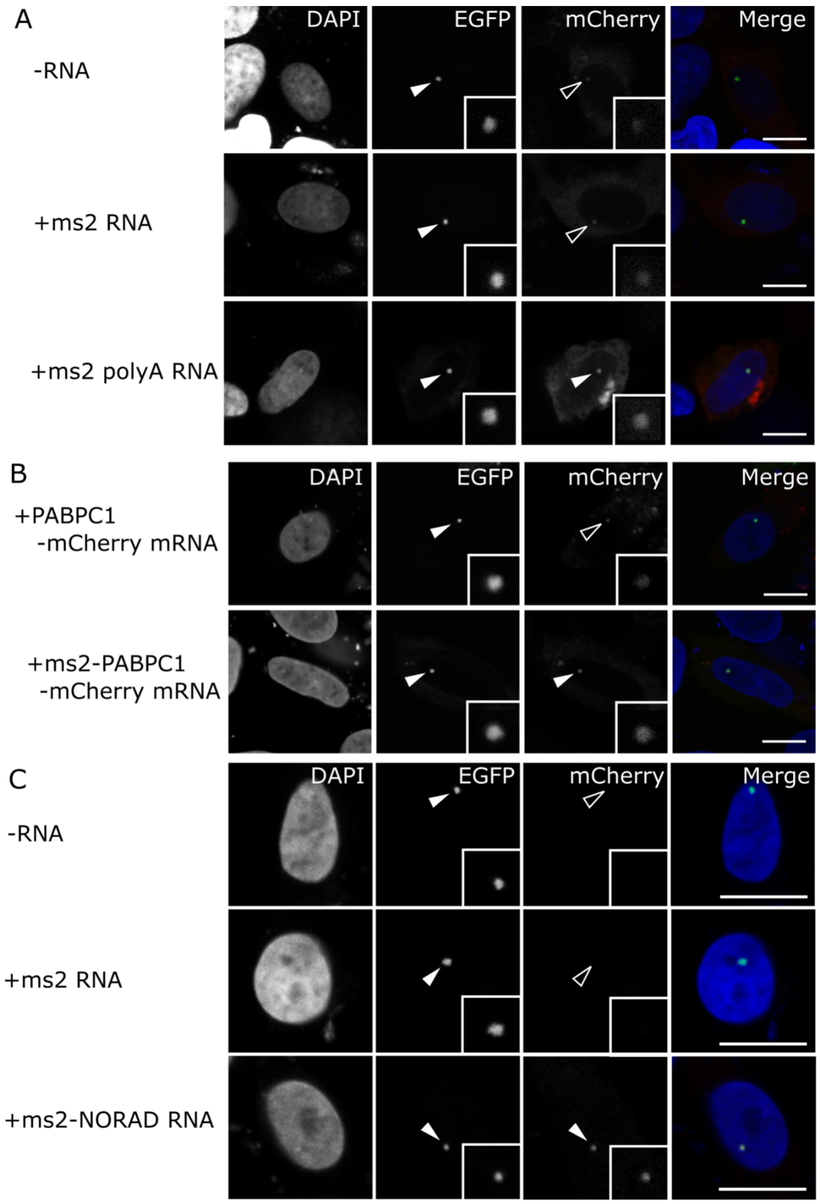

Supplementary Figure 8

(A) rF3H detection of the interaction between an mRNA mimic and PABPC1. The polyadenylated ms2 mRNA mimics recruits and colocalizes with PABPC1 protein at the *lac*O spot (marked as filled arrowheads), but not in the control groups without RNA or without polyadenylation (marked as empty

arrowheads). **(B)** Images of PABPC1-mCherry mRNA and PABPC1 interaction assay with control group, as supplementary for Fig. 2E. **(C)** Complete images of the NORAD ncRNA and PUM2 interaction assay, as supplementary for Fig. 2H. Scale bars stand for 10 μm.

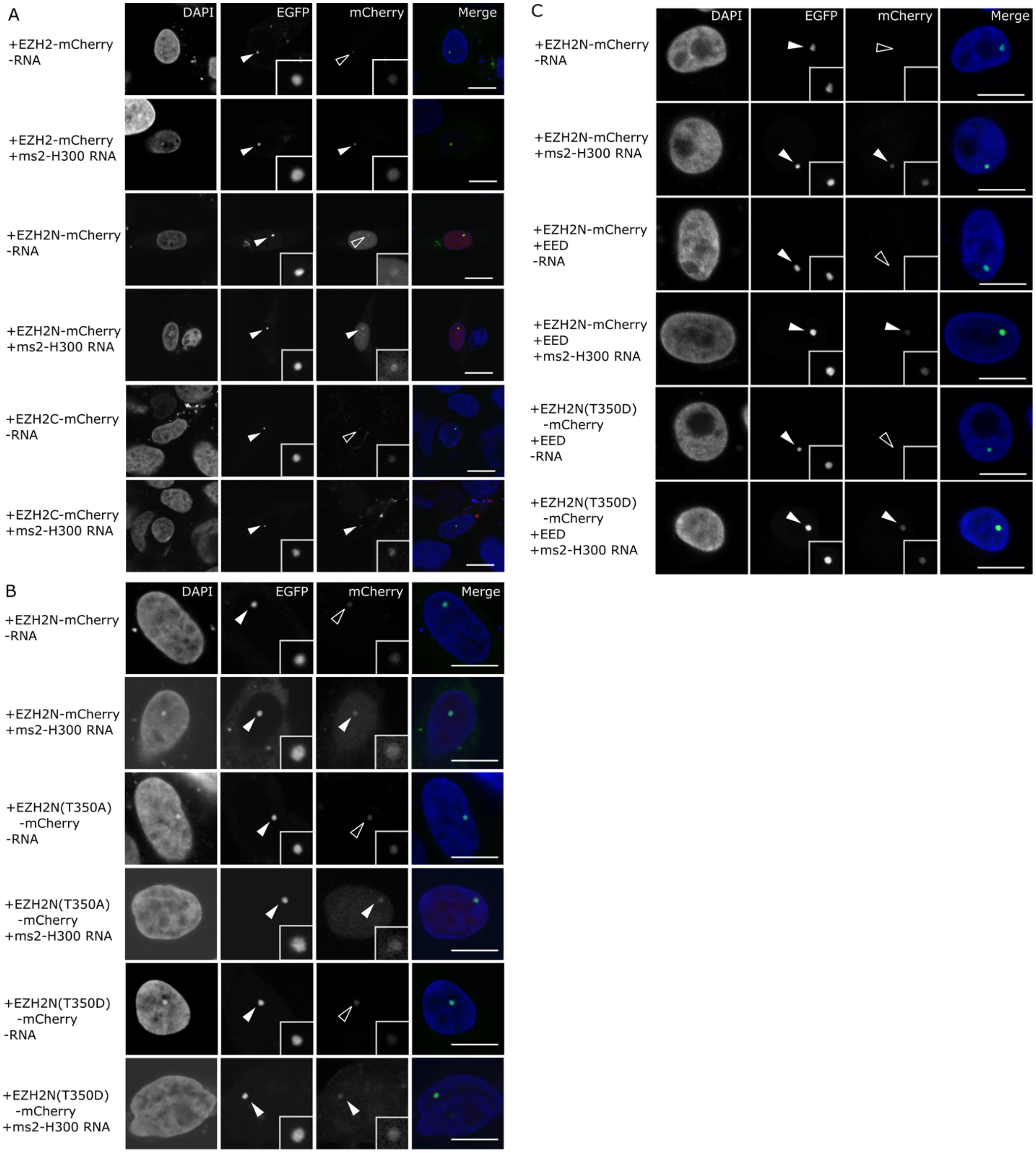

Supplementary Figure 9

(A) rF3H images of the interaction between H300 RNA and EZH2 protein fragments. Both the N- and C-terminal parts of EZH2 are recruited to *lac*O spots by the H300 RNA, but not in absence of the RNA. **(B)** Detection of the interaction between H300 and the EZH2 phosphorylation mimics. A phosphorylation mimic (EZH2 T350D) and an unphosphorylation mimic (EZH2 T350A) were tested, both of which showed interactions with the H300 RNA, but with different binding affinities. Image quantification is shown in Fig. 3G. Scale bars stand for 10 µm, and *lac*O spots are marked by arrowheads (filled arrowheads: enrichment of fluorescence; empty arrowheads: no enrichment of fluorescence). **(C)** The interaction between EZH2 and H300 RNA is promoted by EED protein, which can be further enhanced by T350 phosphorylation, shown here as supplementary for Fig. 3H (filled arrowheads: enrichment of fluorescence; empty arrowheads: no enrichment of fluorescence).

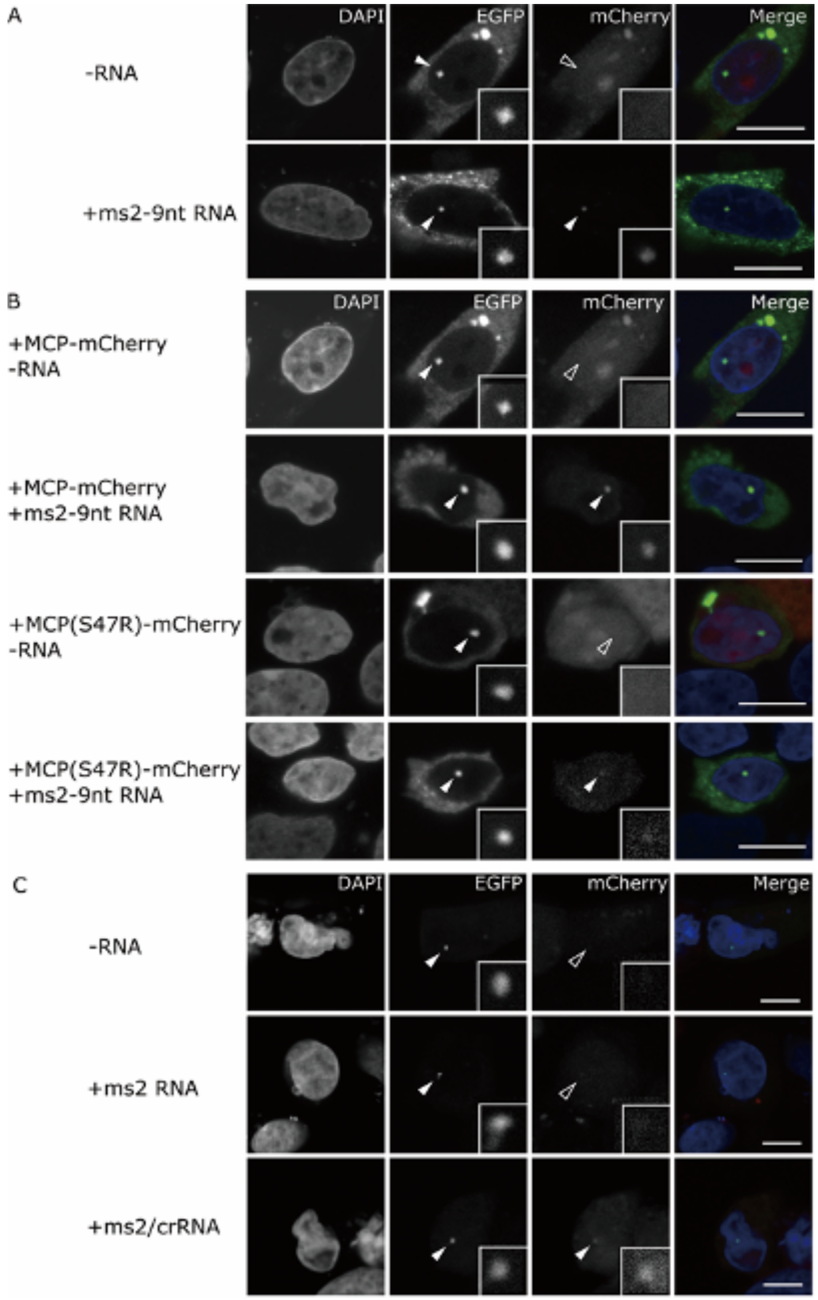

Supplementary Figure 10

(A and **B)** Detection of RNA-protein interaction with PUF RNA trap. ms2 RNA are anchored to nuclear *lac*O spots by PUF RNA trap recognizing a 9 nt RNA sequence, and recruitments of MCP to the same loci are observed (lower panel) but not in control group (upper panel) in (A). rF3H images of the interaction between S47R mutant and ms2 RNA is shown in (B), as a supplementary for Fig. 6D. **(C)** Complete images of ms2 RNA and MCP interaction assay with the dCas13a RNA trap. Scale bars stand for 10 µm.

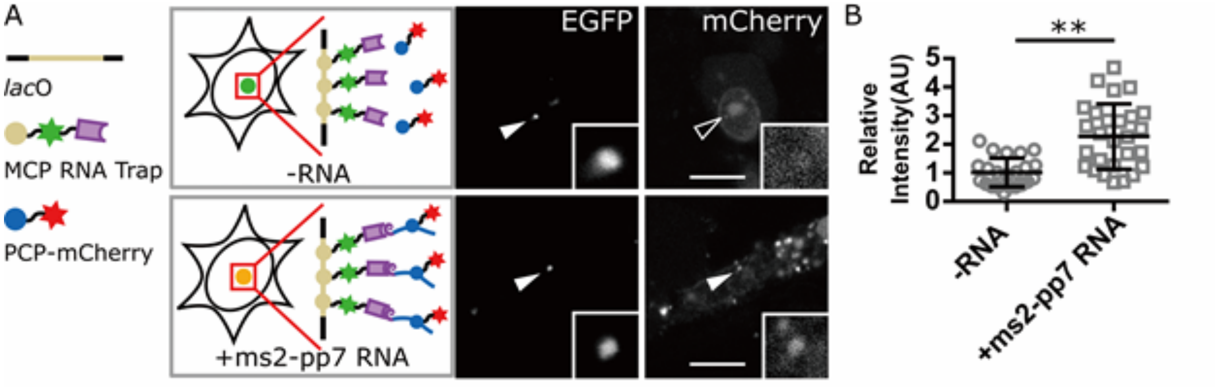

Supplementary Figure 11

Detection of pp7- PCP interaction in living cells. **(A)** Live cell imaging was performed to detect pp7- PCP interaction, and recruitments of PCP-mCherry to *lac*O spots could be observed in the presence of pp7 RNAs, providing the possibility for analyzing dynamical RNA-protein interactions. *lac*O spots are marked with arrowheads. Scale bars represent 10 µm. **(B)** Image quantification reveals a significant enrichment of mCherry labeled PCP proteins at the *lac*O array with the ms2 tagged pp7 RNA, showing a similar detection sensitivity with the fixed samples (data are presented as mean ± S.D., for control and +ms2-pp7 RNA groups, n = 20 and 22, respectively. ** $P < 0.01$).

Supplementary References

1. Izzo,A. (2006) Characterization of MLE RNA helicase, a subunit of the Dosage Compensation Complex (DCC) in Drosophila melanogaster. lmu, 2006.

2. Herce,H.D., Deng,W., Helma,J., Leonhardt,H. and Cardoso,M.C. (2013) Visualization and targeted disruption of protein interactions in living cells. *Nat. Commun.*, **4**, 2660.

3. Plath,K., Fang,J., Mlynarczyk-Evans,S.K., Cao,R., Worringer,K.A., Wang,H., Cruz,C.C. de la, Otte,A.P., Panning,B. and Zhang,Y. (2003) Role of Histone H3 Lysine 27 Methylation in X Inactivation. *Science*, **300**, 131–135.

4. Anton,T., Bultmann,S., Leonhardt,H. and Markaki,Y. (2014) Visualization of specific DNA sequences in living mouse embryonic stem cells with a programmable fluorescent CRISPR/Cas system. *Nucleus*, **5**, 163–172.

5. Daigle,N., Beaudouin,J., Hartnell,L., Imreh,G., Hallberg,E., Lippincott-Schwartz,J. and Ellenberg,J. (2001) Nuclear pore complexes form immobile networks and have a very low turnover in live mammalian cells. *J. Cell Biol.*, **154**, 71–84.

6. Staněk,D. and Neugebauer,K.M. (2004) Detection of snRNP assembly intermediates in Cajal bodies by fluorescence resonance energy transfer. *J. Cell Biol.*, **166**, 1015–1025.

7. Fatscher,T., Boehm,V., Weiche,B. and Gehring,N.H. (2014) The interaction of cytoplasmic poly(A)-binding protein with eukaryotic initiation factor 4G suppresses nonsense-mediated mRNA decay. *RNA*, **20**, 1579–1592.

8. Hafner,M., Landthaler,M., Burger,L., Khorshid,M., Hausser,J., Berninger,P., Rothballer,A., Ascano,M., Jungkamp,A.-C., Munschauer,M., *et al.* (2010) Transcriptome-wide Identification of RNA-Binding Protein and MicroRNA Target Sites by PAR-CLIP. *Cell*, **141**, 129–141.

9. Tichon,A., Gil,N., Lubelsky,Y., Havkin Solomon,T., Lemze,D., Itzkovitz,S., Stern-Ginossar,N. and Ulitsky,I. (2016) A conserved abundant cytoplasmic long noncoding RNA modulates repression by Pumilio proteins in human cells. *Nat. Commun.*, **7**, 12209.

10. Tambe,A., East-Seletsky,A., Knott,G.J., Doudna,J.A. and O'Connell,M.R. (2018) RNA Binding and HEPN-Nuclease Activation Are Decoupled in CRISPR-Cas13a. *Cell Rep.*, **24**, 1025–1036.

11. Gupta,R.A., Shah,N., Wang,K.C., Kim,J., Horlings,H.M., Wong,D.J., Tsai,M.-C., Hung,T., Argani,P., Rinn,J.L., *et al.* (2010) Long non-coding RNA HOTAIR reprograms chromatin state to promote cancer metastasis. *Nature*, **464**, 1071–1076.
